# Robotic Prostatectomy Has a Superior Outcome in Larger Prostates and PSA Density Is a Strong Predictor of Biochemical Recurrence

**DOI:** 10.1155/2014/763863

**Published:** 2014-12-15

**Authors:** S. Bishara, N. Vasdev, T. Lane, G. Boustead, J. Adshead

**Affiliations:** Department of Urology, Hertfordshire and South Bedfordshire Robotic Urological Cancer Centre, Lister Hospital, Coreys Mill Lane, Stevenage, Hertfordshire SG1 4AB, UK

## Abstract

*Objectives*. The aims of this study were to compare the outcomes of robotic assisted laparoscopic prostatectomy (RALP) between patients who had larger (≥75 g) and smaller (<75 g) prostates and to evaluate the performance of PSA density (PSAD) in determining the oncological outcome of surgery. *Methods and Materials*. 344 patients who underwent RALP at a single institution were included in the study. Preoperative risk factors and postoperative, oncological outcomes, erectile function, and continence status were recorded prospectively. *Results*. During a mean follow-up of 20 months, biochemical recurrence (PSA > 0.2) was observed in 15 patients (4.3%). Prostate size ≥75 g was associated with lower Gleason score on final pathology (*P* = 0.004) and lower pathological stage (*P* = 0.02) but an increased length of hospital stay (*P* = 0.05). PSAD on binary logistic regression independently predicted biochemical recurrence (BCR) when defined as postoperative PSA >0.1 (*P* = 0.001) and PSA >0.2 (*P* = 0.039). In both instances PSA was no longer a significant independent predictor. *Conclusions*. RALP in large prostates (≥75 g, <150 g) is as safe as RALP in smaller prostates and is associated with a lower pathological grade and stage. Higher PSAD is independently associated with BCR and is superior to PSA as a predictor of BCR after RALP.

## 1. Introduction

A number of studies have evaluated the risk factors for biochemical recurrence after radical prostatectomy [1, 2]. PSA, age, Gleason score, and clinical stage have been demonstrated to be independent predictors of outcome. Preoperatively, however, there is considerable risk of under staging of disease, even with the use of multiparametric MRI and increased sampling of the prostate gland on biopsy. A large prostate gland can be surgically challenging at robotic prostatectomy and unsurprisingly is associated with increased operative time, transfusion rate, and risk of postoperative complications [3] but despite this, when evaluated as a prognostic factor it is associated with a superior oncological outcome in terms of positive margin rate and biochemical recurrence [2, 4]. A logical extension of the utilisation of PSA in the preoperative evaluation of patients being considered for RALP is to combine it with prostate size to calculate a PSA density. PSA density is an established prognostic factor that has been widely evaluated but often unused in the diagnostic pathway, particularly as an accurate assessment of prostate size is dependent on MRI or transrectal U/S and therefore the patient is well on the way along the diagnostic pathway before the PSAD is determined. Thus it is unlikely to alter investigative strategy, though it may be a readily available determinant of therapeutic outcomes. Typically PSAD cutoffs of greater than 0.15 have been used to prognosticate higher risk disease in patients undergoing prostatectomy [5].

The principle aims of this study were to record the differences in outcome between smaller <75 g and larger ≥75 g prostates and to test the hypothesis that PSAD is superior to PSA in determining the outcome of RALP for prostate cancer.

## 2. Methods

### 2.1. Patients

A prospective database of all patients undergoing radical robotic prostatectomy between 2008 and 2013 at a single centre was created. Preoperative status, staging investigations, intraoperative parameters, postoperative outcomes, complications, follow-up biochemical or pathological recurrence, and erectile and continence function were recorded. The patient follow-up schedule was every 3 months for the first year and every 6 months for the second year and yearly thereafter.

### 2.2. Categorisation and Statistical Analysis

The prostate size was defined according to the pathological specimen weight. PSAD was defined as prostate size divided by the preoperative PSA level. Upstaging was defined as increase in T numerical denomination between clinical and pathological staging. Postoperative complications were defined according to the Clavien Scale and split into two groups: minor (Clavien 1 and 2) and major (Clavien 3 and 4) complications. Continence was classified according to daily pad usage (no pads, 1 pad, 2 pads, 3 pads, and 4 or more pads). Erectile function was categorised in four groups (normal spontaneous erections, good erections with PDE5, partial erections with PDE5, and no erections with PDE5, requiring caverject injections). For comparison of the difference in outcomes according to prostate size, patients were divided into two groups: <75 g and ≥75 g. The difference between the two groups was compared using an unpaired *t*-test for continuous variables and the Fisher exact test for proportions.

### 2.3. Evaluation of PSA Density

A multivariate model to predict margin positivity and biochemical recurrence at the PSA >0.1 level and PSA > 0.2 level was created using the standard preoperative factors: PSA, biopsy Gleason score (<7, 7, >7), clinical stage (T1, T2, T3), and age in addition to PSAD. Analysis was with binary logistic regression.

A second method using Cox proportional hazards was used to evaluate the effect of adding PSAD to a model that contained PSA, biopsy Gleason score, and clinical stage. The strength of these models and the effect of adding PSAD were evaluated by the Chi squared test. In all cases clinical significance was taken as *P* ≤ 0.05. Statistical analysis and graphing were carried out by IBM SPSS version 22. Patients were excluded from a statistical analysis when a prerequisite parameter was missing.

## 3. Results

344 patients were included in the study. 296 patients had a prostate weight of <75 g and 48 a prostate weight of ≥75 g. Mean follow-up of patients amongst the two groups was similar (1.7 years >75 g, 1.6 years ≥75 g, ns).

Preoperative staging (Table 1): patients with prostates ≥75 g were slightly older (63.2 versus 61.4 years, *P* = 0.05) than those with prostates <75 g. Otherwise, there was no significant difference in the preoperative risk factors: PSA, clinical stage, and biopsy Gleason score.

Pathology and oncological outcomes of RALP (Table 2): larger prostates (≥75 g) were more likely to be staged as T1 pathological stage (8.3% versus 1.4%, *P* = 0.02), less likely to be upstaged on final histology (58.3% versus 75.3%, *P* = 0.02), and more likely to have lower grade disease on final histology (41.7% versus 26.4%, *P* = 0.04). There was, however, no difference between these two groups in terms of type of nerve sparing procedure, lymph node dissection rate, positive surgical margin, and biochemical recurrence rate.

Postoperative outcomes and complications of RALP: in Table 3 larger prostates (≥75 g) were associated with a longer operative time (255 versus 222 min, *P* = 0.002), greater blood loss (349 versus 219 mL, *P* = 0.0002), and blood transfusion requirements (0.01 versus 0.13 units per operation, *P* = 0.006). Larger prostates (≥75 g) were associated with a longer postoperative stay (2.82 versus 2.26 days, *P* = 0.04, and longer postoperative catheter time (10.3 versus 9.2 days, *P* = 0.05).

**Figure 1:**
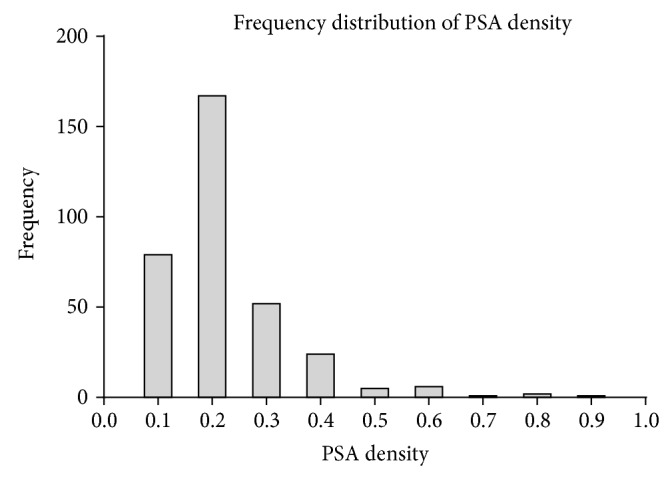


**Figure 2:**
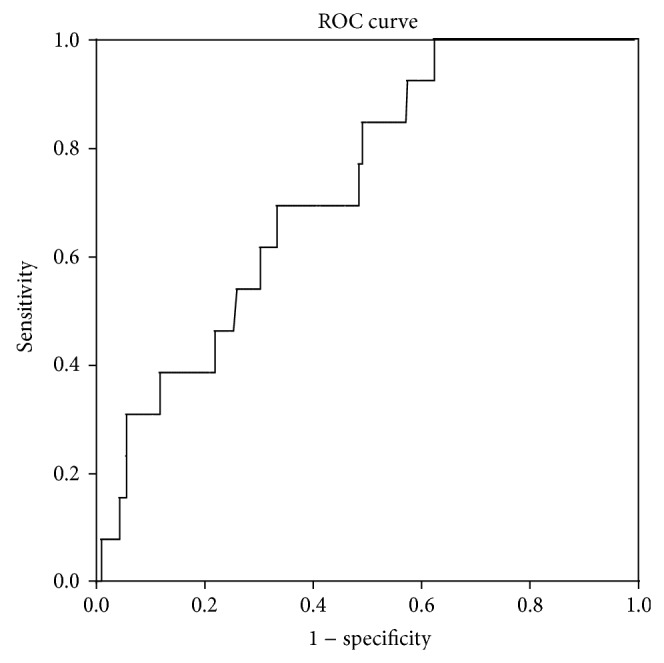
ROC curve for the prediction of biochemical recurrence (PSA > 0.2) by PSA density. *R* ^2^ = 0.73.

PSAD: mean PSAD was 0.17 ranging from 0.022 to 0.875 and has a positive skew distribution as shown in Figure 1. PSAD alone was predictive of biochemical recurrence as shown in Figure 2, with area under the receiver operated curve of 0.73. A PSAD of >0.136 corresponds to an 85% sensitivity and 51% specificity for detecting biochemical recurrence. The comparable areas under the ROC for predicting biochemical recurrence were 0.69 for PSA, 0.52 for clinical stage, and 0.62 for transrectal biopsy Gleason sum score.

On multivariate analysis of preoperative staging parameters and PSAD, using binary logistic regression (*n* = 309), Gleason sum score was found to be the only independent predictor of margin positivity (*P* = 0.013) (Table 4). BCR at PSA >0.1 was only predictable by PSAD (*P* = 0.001). Likewise BCR at PSA >0.2 was only predictable by PSAD (*P* = 0.039), despite the inclusion of PSA in both models.

A further analysis using Cox proportional hazards was carried out, where PSAD was added to a model containing Gleason score, clinical stage, and PSA. The effect of adding PSAD to the model increased the predictive power for BCR at PSA >0.1 (Chi square increase from 12.021, df 4, to 19.084, df 5, *P* = 0.017) and BCR at PSA >0.2 (Chi square increase from 9.832, df 4, to 13.15, df 5, *P* = 0.110).

## 4. Discussion

Whilst larger prostates present a technical challenge at robotic prostatectomy and are associated with a longer operative time [6], greater blood loss, and increased postoperative complications [3, 7], they are associated with a superior oncological outcome in terms of reduced extraprostatic disease, reduced positive margin rate [8–14], and decreased biochemical recurrence rate [3]. In this cohort there was no significant difference between larger prostates (≥75 g) and smaller prostates (<75 g) in terms of the preoperative staging; however, the two groups were markedly different postoperatively. A smaller prostate was associated with a higher Gleason grade and a higher pathological stage at robotic prostatectomy. Smaller prostates were more likely to be upstaged. Whilst this might be counterintuitive, in that a larger prostate is relatively undersampled at TRUS and biopsy, the increased sensitivity of PSA for detecting prostate cancer in smaller glands (as quantified by PSAD) given the lower PSA signal from BPH seems to supervene.

There was a small but statistically significant increase in the length of postoperative stay (0.6 days) and catheter time (1 day) in prostates ≥75 g but no difference between the two groups in terms of postoperative complications, indicating that robotic prostatectomy in large prostates is a safe procedure.

PSAD was first proposed as an enhancement to PSA as a prognostic factor in 1992 by Benson et al. [15] as a means of distinguishing prostate cancer from BPH. Since that time, a number of studies have examined PSAD as a preoperative risk factor in radical prostatectomy [16–20] and more recently robotic prostatectomy [2, 4, 21].

Two previous studies in RALP have demonstrated PSAD as an independent predictor of PSM [4, 16]. In this study, PSAD was not found to be independently predictive of PSM when PSA, biopsy Gleason score, and clinical stage were included in a multivariate model.

This is only the second study which has examined the prognostic power of PSAD in RALP for BCR after the recently published study by Hashimoto et al. [2]. In their study, PSAD was associated with biochemical recurrence but it was not an independent risk factor when PSA was used. In this study PSAD displaces PSA as an independent risk factor for biochemical recurrence. The superiority of PSAD over PSA in the determination of biochemical recurrence in open prostatectomy has previously been examined. Radwan et al. [17] found in their cohort of 1327 radical prostatectomies that a model incorporating PSA density rather than PSA was superior in predicting BCR. Likewise Freedland et al. in 2002 [19] found that PSAD was superior to PSA in determining BCR. However, in a later publication in 2003 [20] this difference was deemed to be clinically insignificant. This is the first paper to demonstrate the superiority of PSAD as a prognostic factor in RALP, in accordance with the findings of Radwan et al. in open prostatectomy [17].

This study has a relatively short median follow-up of 20 months and low recurrence rate of 4.3% at a mean follow-up of 20 months. By comparison a recently published study yielded recurrence rates of 12.8% at median follow-up of 28 months [2]. Despite the low recurrence rate which reduces statistical power, PSAD was able to demonstrate superiority to PSA in detecting BCR at PSA >0.2. The overall number of patients in this study is less than Radwan and Kundu studies which included 1327 and 1280 patients, respectively, albeit undergoing open prostatectomy, and the number of patients with prostates ≥75 g amounted to only 48.

A further weakness of this study is that the pathological specimen weight was used to calculate PSAD rather than volume on preoperative TRUS or MRI, as these were not always available on account of the initial staging investigations being performed at other hospitals. PSAD has most utility as a preoperative marker; however, TRUS volume and MRI volume demonstrate a strong concordance with prostate specimen weight [22, 23].

In conclusion PSAD is superior to PSA in predicting biochemical recurrence after robotic prostatectomy and should be taken into consideration in the analysis of outcome in any RCT examining treatment alternatives in localised prostate cancer, as it is likely to influence the risk benefit ratio.

## Figures and Tables

**Table 1:** Preoperative staging.

Prostate size	<75 g, *n* = 296		>75 g, *n* = 48		*P*
	Mean/proportion	sd/%	Mean/proportion	sd/%	
PSA	8.21	5.3	9.45	5.3	ns
Age	61.4	5.9	63.2	5.7	0.05
Biopsy stage					
T1	139/261	53.3%	25/48	52%	ns
T2	120/261	46%	21/48	43.8%	ns
T3	2/261	0.7%	2/48	4.2%	ns
Biopsy grade					
G6	120/287	41.8%	24/48	50%	ns
G7	147/287	51.2%	21/48	43.8%	ns
G8–10	20/287	6.9%	3/48	6.3%	ns

**Table 2:** Pathology and oncological outcomes of RALP.

Prostate size	<75 g, *n* = 296		>75 g, *n* = 48		*P*
	Mean/proportion	sd/%	Mean/proportion	sd/%	

Prostate weight	47.48	12.29	97.23	20.7	<0.0001
Nerve spare					
Bilateral NS	92/293	31.4%	15/47	31.9%	ns
Unilateral NS	91/293	31.1%	11/47	23.4%	ns
WLE	100/293	34.1%	21/47	44.6%	ns
Lymph node dissection	34/296	11.5%	4/48	8.3%	ns
Pathological stage					
T1	4/296	1.4%	4/48	8.3%	0.02
T2	226/296	76.4%	32/48	66.7%	ns
T3	64/296	21.6%	12/48	25%	ns
T4	1/296	0.3%	0/48	0%	ns
Positive margin	81/296	27.3%	12/48	25%	ns
Upstage	196/260	75.3%	28/48	58.3%	0.02
SVI^a^	14/296	4.7%	5/48	10.4%	ns
Pathological grade					
G6	78/296	26.4%	20/48	41.7%	0.04
G7	189/296	63.9%	20/48	41.7%	0.004
G8–10	29/296	9.8%	8/48	16.7%	ns
Biochemical recurrence	14/296	4.7%	1/48	2.1%	ns

^a^SVI: seminal vesicle involvement.

**Table 3:** Postoperative outcomes and complications of RALP.

Prostate size	<75 g, *n* = 296		>75 g, *n* = 48		*P*
	Mean/proportion	sd/%	Mean/proportion	sd/%	
Postop stay/days	2.262	1.62	2.822	2.229	0.04
Catheter time/days	9.166	3.62	10.29	4.177	0.05
Number of pads/day 6 m^a^	0.482	0.781	0.540	0.802	ns
Number of pads/day 12 m^a^	0.2981	0.630	0.117	0.332	ns
ED score 6 m^*β*^	2.354	0.9377	2.441	0.823	ns
ED score 12 m^*β*^	2.129	1.049	2	1.061	ns
Operative time	221.7	66	255.4	85.46	0.002
Recorded blood loss	219.4	189.7	348.5	310.3	0.0002
Blood transfusion requirements	0.011	0.1385	0.13	0.62	0.006
Complications					
Clavien 1-2	20/298	6.7%	7/48	14.5%	0.07
Clavien 3-4	13/298	4.3%	4/48	8.3%	ns

^a^Daily pad usage was scored as 0 for no pads, 1 for one pad, 2 for two pads, 3 for three pads, and 4 for four or more pads.

^*β*^Erectile dysfunction was scored as 0 for normal spontaneous erections, 1 for good erections with PDE5, 2 for partial erections with PDE5, and 3 for no erections with PDE5, requiring caverject injections.

**Table 4:** Multivariate binary logistic regression of preoperative risk factors and outcome of RALP.

Preoperative risk factors	Mean	Margin +ve		BCR PSA > 0.1		BCR PSA > 0.2	
		Coefficient	*P* value	Coefficient	*P* value	Coefficient	*P* value
Age	61.6	−0.029	ns	0.419	ns	0.047	ns
PSA	8.146	0.051	ns	2.983	ns	−0.108	ns
PSA density	0.17	0.526	ns	11.65	0.001	9.022	0.039
Gleason score (<7, 7, and >7)	143, 175, 25	0.63	0.013	3.314	0.069	−0.146	ns
Clinical stage (T1, T2, and T3)	161, 138, 5	−0.164	ns	1.832	ns	0.322	ns
